# Gender specific association between the use of complementary and alternative medicine (CAM) and alcohol consumption and injuries caused by drinking in the sixth Tromsø study

**DOI:** 10.1186/s12906-018-2301-y

**Published:** 2018-08-13

**Authors:** Kristina Sivertsen, Marko Lukic, Agnete E. Kristoffersen

**Affiliations:** 1 Department for drugs – and addiction treatment and A-larm Norway, Hospital of Southern Norway, Kristiansand, Norway; 2 Department of Community Medicine, Faculty of Health Sciences, UiT The Arctic University of Norway, Tromsø, Norway; 3 National Research Center in Complementary and Alternative Medicine (NAFKAM), Department of Community Medicine, Faculty of Health Sciences, UiT The Arctic University of Norway, Tromsø, Norway

**Keywords:** Complementary and alternative medicine, CAM, Herbal medicine, Self-treatment, Alternative medical practitioner, Alcohol consumption, Alcohol-related injuries, Cross-sectional study, The Tromsø study

## Abstract

**Background:**

Alcohol is consumed almost worldwide and is the most widely used recreational drug in the world. Harmful use of alcohol is known to cause a large disease-, social- and economic burden on society. Only a few studies have examined the relationship between CAM use and alcohol consumption. To our knowledge there has been no such research in Norway. The aim of this study is to describe and compare alcohol consumption and injuries related to alcohol across gender and different CAM approaches.

**Methods:**

The data used in this study is based on questionnaire data gathered from the sixth Tromsø Study conducted between 2007 and 2008. Information on CAM use and alcohol consumption was available for 6819 women and 5994 men, 64.8% of the invited individuals. Pearson chi-square tests and independent sample t-tests were used to describe the basic characteristics of the participants and to calculate the differences between men and women regarding these variables. Binary logistic regression analyses were used to investigate the associations between the different CAM approaches and alcohol consumptions and injuries caused by drinking.

**Results:**

Women who drank alcohol more than once a month were more likely to have applied herbal or “natural” medicine and self-treatment techniques (meditation, yoga, qi gong or tai-chi), compared to those who never drank, and those who only drank monthly or less. For women, an association was also found between having experienced injuries caused by drinking and use of self-treatment techniques and visit to a CAM practitioner. No association was found between amount of alcohol consumed and use of CAM approaches. For men, an association was found between injuries caused by drinking and use of herbal or “natural” medicine.

**Conclusion:**

The findings from this cross-sectional study suggests that women who drink frequently are more likely to use “natural” medicine and self-treatment techniques. Both men and women who had experienced injuries because of their drinking were more likely to have used CAM approaches.

## Background

Alcohol is consumed almost worldwide and is the most widely used recreational drug in the world [1]. However, alcohol consumption varies across countries and cultures and there are wide variations within global estimates [1, 2]. The highest levels of alcohol consumption are found in Europe (10.9 l per inhabitant over the age of 15 (15+)), followed by the Americas (8.4 l) the Western Pacific Region (6.8 l) and Africa (6.0 l). The lowest level is found in South-East Asia, especially in the Eastern Mediterranean (0.7 l) [1]. In Norway, people drink on average six litres of pure alcohol a year [3]. When unrecorded consumption, such as border trade and tax-free commerce is included, the number is estimated to be about 7.7 l per inhabitant (15+) [1, 4]. In the Norwegian city Tromsø, the general alcohol consumption is found to be relatively low, reflecting the modest alcohol consumption in Norway [5].

Harmful use of alcohol is known to cause a large disease-, social- and economic burden on society [1, 6]. Despite varying estimates of alcohol use, most countries show substantial disease and death rates attributed to alcohol consumption [1, 2]. Harmful alcohol use is among the five leading risk factors for disease, disability and preventable death [1, 7, 8], and contributes to 7.4% of total diseases burden for men and 3% for women [1].

Complementary and alternative medicine (CAM) is used worldwide, but have often been an underestimated part of health care. More countries are now increasingly recognizing and accepting CAM’s contribution to individual’s health and well-being and its contribution to health care [9]. In the last 30 years there has been an increasing interest and use of CAM, particularly in Western societies [10–12].

The definition of CAM differ across countries and organizations. According to the World Health Organization (WHO), CAM is defined as a broad spectre of health services that are not incorporated in a countries traditional health care system and is not part of public health services [9]. In Norway, CAM providers offers treatment both as an alternative to, and complementary to conventional treatment. As such, the CAM providers offers therapies that are not usually a part of the public health care system and are paid by out of pockets payments [13].

CAM is often used by people suffering from chronic conditions or life-threatening and serious illness such as cancer [11, 13], chronic pain [14, 15], mental disorders [16] and/or in situations when conventional treatment options are limited [15]. However, motives for use also include a range of other reasons, including using CAM as preventive therapies, CAM being more congruent with their personal belief system, CAM’s ability to provide hope, the notion that CAM offers a more holistic view of health care, the therapeutic value of CAM, more emphasis on patient control, and a perception that CAM practitioners offers a more supportive role compared to conventional health care personal [10].

CAM use is believed to be closely associated with sociodemographic variables such as female gender, young to middle age, middle to high income, high level of education and poorer self-perceived health [10, 17–19]. According to a Norwegian survey, close to half of the female participants reported to have used some kind of CAM, while one out of four male participants reported the same [20]. Gender differences in use of CAM has also been found in other Norwegian [20, 21] and international studies [10, 22].

Although there has been focus on a range of sociodemographic characteristics associated with use of CAM, only a few studies have examined the relationship between CAM use and alcohol consumption. They indicated that use of CAM is associated with different level of alcohol use [23–26]. Having consumed alcohol in one’s life but not being a heavy drinker [27] as well as less frequent alcohol consumption [28] was associated with CAM use. The findings have, however, been ambiguous. Another study found an inverse relationship between alcohol consumption and CAM use [29], while several other studies failed to find any significant association between the two [30–32].

To our knowledge, there has been no research comparing alcohol consumption between users and non-users of CAM in Norway. Since both alcohol patterns and use of CAM is strongly associated with gender, the aim of this study is to describe and compare gender specific alcohol consumption and injuries related to alcohol across gender and use of different CAM approaches.

## Methods

### The study population

The Tromsø Study is a population-based, prospective study of a range of health related issues and is considered a great resource for surveillance of risk factors and disease in the population [33]. This study is based on the sixth Tromsø Study conducted between October 2007 and December 2008. The invited population came from four groups: people who participated in the second visit in the fourth Tromsø study conducted in 1994/1995, a 10 % random sample of people aged 30–39, all individuals aged 40–42 and 60–87 and a 40% random sample of people aged 43–59 years, all residing in the municipality of Tromsø [34].

An invitation containing information and a four-page questionnaire (Q1) was sent by mail to the participants within 2 wks of a suggested appointment for a physical examination [35]. A total of 19,762 people between 30 and 87 years were invited [36], with a participation rate of 65.7% (12,981 participants).

Q1 was filled out at home and brought to the examination. Q1 included questions on various health issues, symptoms and diseases, use of medication and healthcare services, disability, employment, income, lifestyle, and reproduction. The second questionnaire (Q2), of 28 pages, was handed out during the examination, and the participants could either fill it out at the spot or return it later in prepaid postage envelopes. Q2-data was available for 95.8% of the participants who filled out Q1, and contained follow-up questions of topics covered in Q1 [36].

**Fig 1:**
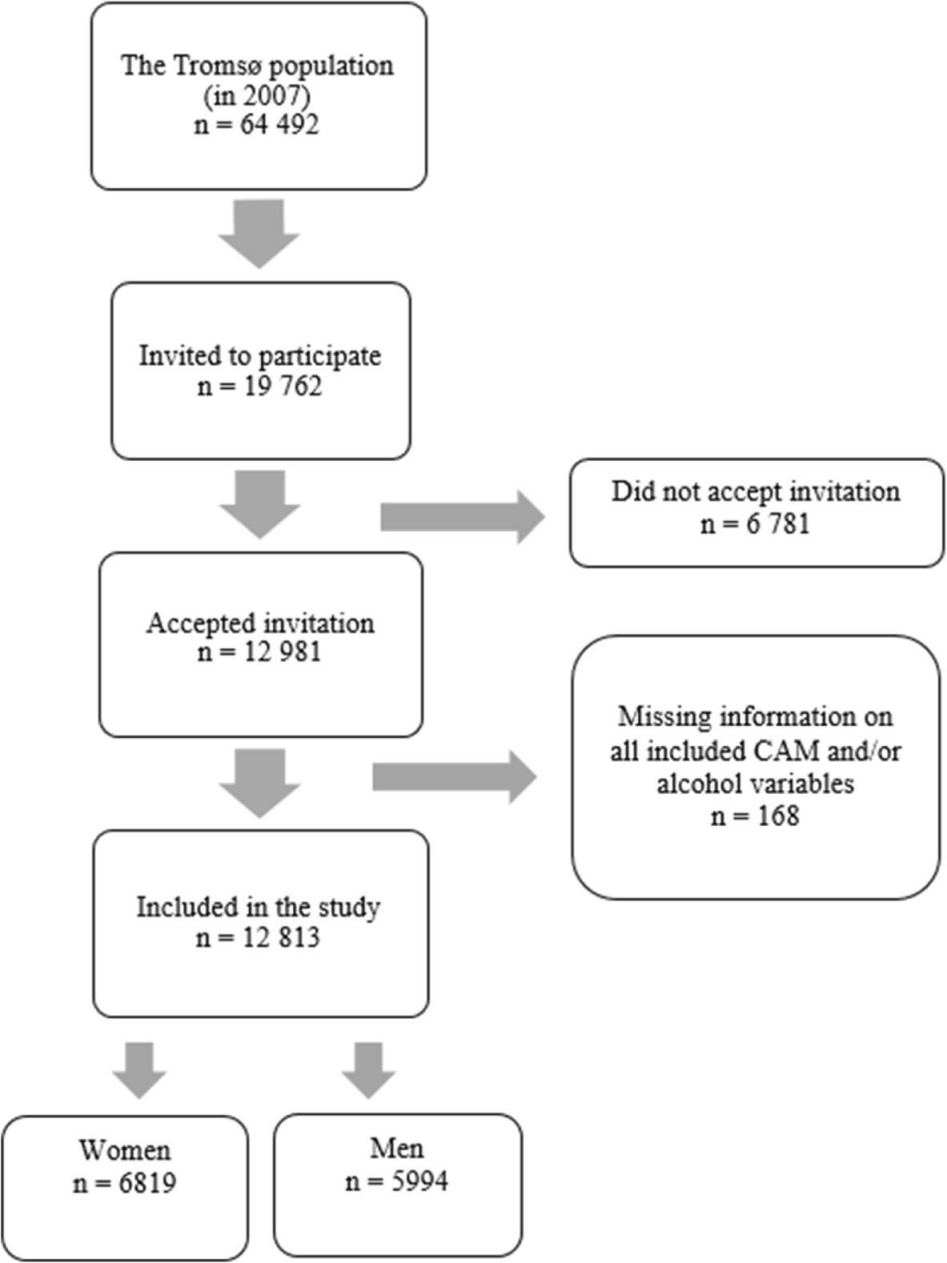
Flow chart of the studied population

As shown in Fig. 1, we excluded participants who refrained from answering any of the three included CAM questions and/or any of the three included alcohol questions (*n* = 168). A total of 12,813 participants (64.8% of the invited individuals), 6819 women and 5994 men were included in the analyses.

### Assessment of CAM use and alcohol consumption

Use of alcohol is based on self-reported consumption of alcohol gathered from Q1 [37] and Q2 [38]. From Q1, the two following questions were used: Firstly: “*How often do you drink alcohol?*” with the response options: “*Never*”, “*Monthly or more infrequently*”, “*2-4 times a month*”, “*2-3 times a week*”, “*More than 3 times a week*”. The first category “*Never*” was used as the reference category for all analyses including alcohol frequency. Secondly, “*How many units of alcohol (a beer, a glass of wine or a drink) do you usually drink when you drink alcohol?*”, with five possible answers: “*1–2*”, “*3–4*”, “*5–6*”, “*7–9*”, “*10 or more*”. The categories with highest level of consumption had few respondents and were collapsed into the category “*5 or more*” as five or more drinks in one occasion is defined as heavy episodic drinking and have been associated with increased risk of harm [1, 39, 40]. The first option, “*1–2*” units, was set as the reference category. From Q2, the following question was included in the analyses: “*Have you or someone else been injured because of your drinking*?”, with “*Never*”, “*Yes, but not in the last year*” and “*Yes, during the last year*” as the response options. Due to few respondents in the two last categories, these were merged into one “*Yes*”-category. “*Never*” was set as the reference category.

In order to get information on the use of CAM, three questions were analysed separately. “*Have you during the past year visited: Alternative medical practitioner (homeopath, acupuncturist, foot zone therapist, herbal medical practitioner, laying of hands practitioner, healer, clairvoyant, etc.)*”, with the two options, “*Yes*” and “*No*”. The participants were also asked: “*In the last 12 months have you used meditation, yoga, qi gong or tai-chi as self-treatment*?” and “*In the last 12 months have you used herbal or “natural” medicine*?” with “*Yes*” and “*No*” as response options. The different CAM variables were not mutually exclusive, as many of CAM users tend to use more than one approach.

### Statistical methods

Pearson chi-square tests and independent sample t-tests were used to describe the basic characteristics of the participants and to calculate gender differences regarding these variables. The association between alcohol consumption and the use of CAM was investigated in binary logistic regression models. Each of the CAM approaches (visit to alternative practitioner, use of herbal medicine, and self-treatment) were dichotomised to yes/no and used as a dependent variable in the regression model. We calculated odds ratios (OR) with 95% confidence interval (CI) of having used the three CAM approaches according to alcohol exposure. All the analyses were stratified according to gender. Level of education, household income, age and self-reported health status were included as independent variables in all the adjusted models.

Analyses for each of the outcomes were adjusted for the factors that could have influenced the association between alcohol consumption and the use of CAM [4, 10, 17, 41, 42]. These include level of education (primary, 1–2 years secondary school, vocational school, high secondary school (A-level), college/university less than 4 years, and college/university 4 years or more), household income (low income (< 200,000 NOK/ 20,000 €), low middle income (201,000–400,000 NOK/20,100–40,000 €), high middle income (401,000–700,000 NOK/40,100–70,000 €), high income (> 701,000 NOK/70,100 €)), age (continuous), and self-reported health status (bad, neither good nor bad, and good).

All the analyses were carried out using the statistical program IBM SPSS, version 24. *P*-values <0.05 were considered statistically significant for all conducted analyses.

## Results

### Characteristics of the studied participants

The studied population consisted of 6819 women and 5994 men, with the mean age of 57.3 (SD12.9) and 57.4 (SD12.3), respectively. Gender differences was found in regards to education level, household income, self-reported health status, alcohol consumption levels, injuries caused by drinking, and use of all CAM approaches (Table 1). Most of the participants (62%) had middle to high income (> 40,000 €) and good health (66%) and one third of the participants had university education (Table 1).

**Table 1:** Basic characteristics of the studied participants

	Total *n* = 12813^a^ (%)	Men *n* = 5994^a^ (%)	Women *n* = 6819^a^ (%)	*P*-value
Percentage women	53.2			
Age, mean (SD)	57.4 (12.6)	57.4 (12.3)	57.3 (12.9)	0.717^b^
Education, n (%)				
Compulsory	3596 (28.4)	1478 (25.0)	2118 (31.5)	
Middle level	4241 (33.6)	2096 (35.4)	2146 (31.9)	
College/University	4809 (38.0)	2349 (39.7)	2460 (36.6)	< 0.000^c^
Household income, n (%)				
Low to middle income < 400,000 NOK/40,000 €)	4569 (38.5)	1842 (32.0)	2727 (44.6)	
Middle to high income (401,000–700,000 NOK/40,100–70,000 €)	4199 (35.4)	2235 (38.9)	1964 (32.1)	
High income (701,000 NOK / 70,100 € or more)	3093 (26.1)	1668 (29.0)	1425 (23.3)	< 0.000^c^
Self-reported health status, n (%)				
Very bad or bad	686 (5.4)	279 (4.7)	407 (6.0)	
Neither good nor bad	3633 (28.6)	1671 (28.1)	1962 (29.1)	
Good or excellent	8386 (66.0)	4004 (67.2)	4382 (64.9)	0.001^c^
Alcohol frequency of use, n (%)				
Never	1413 (11.2)	454 (7.6)	959 (14.3)	
Monthly or more infrequently	3633 (28.7)	1545 (26.0)	2088 (31.1)	
2–4 times a month	4834 (38.2)	2481 (41.7)	2353 (35.0)	
2–3 times a week	2155 (17.0)	1125 (18.9)	1030 (15.3)	
More that 3 times a week	634 (5.0)	342 (5.8)	292 (4.3)	< 0.000^c^
Units of alcohol consumed when drinking, n (%)				
1–2 units	7095 (63.3)	2858 (52.3)	4237 (73.8)	
3–4 units	3020 (26.9)	1754 (32.1)	1266 (22.0)	
5 or more units	1091 (9.7)	852 (15.6)	239 (4.2)	< 0.000^c^
Injuries because of drinking, n (%)				
Never	10,882 (93.5)	4937 (89.4)	5945 (97.2)	
Yes	752 (6.5)	583 (10.6)	169 (2.8)	< 0.000^c^
Overall use of CAM modalities, n (%)	3730 (33.6)	1259 (24.1)	2471 (42.1)	< 0.000^c^
Alternative medical pratictioner^d^	1423 (11.9)	428 (7.6)	995 (15.9)	< 0.000^c^
Herbal or ‘natural’ medicine^e^	2677 (23.0)	937 (17.1)	1740 (28.3)	< 0.000^c^
Self-treatment^f^	590 (5.0)	107 (1.9)	483 (7.8)	< 0.000^c^

^a^ Due to missing responses on the individual questions, not all number add up to total number of participants. ^b^ Independent sample t-test. ^c^ Pearson Chi-square test ^d^ Answered yes to: Have you during the past year visited: An alternative medical practitioner (homeopath, acupuncturist, foot zone therapist, herbal medicine practitioner, laying on of hands practitioner, healer, clairvoyant etc.)? ^e^ Answered yes to: In the last 12 months have you used herbal or “natural” medicine? ^f^ Answered yes to: In the last 12 months have you used meditation, yoga, qi gong or tai-chi as self-treatment?

More women (11%) than men (8%) were teetotallers. Most of the participants (68% of the men and 66% of the women) drank less than five times a month. Only 6% of the men and 4% of the women drank more than 3 times a week. Most of the women (74%) and half of the men (52%) drank 1–2 unites when drinking alcohol. Very few women (4%) drank more than 4 units when drinking (Table 1). More women (42%) than men (24%) had used CAM. Most of the participants had used herbal or “natural” medicine (23%) followed by alternative medical practitioner (12%) and self-treatment with meditation, yoga, qi gong or tai-chi (5%) (Table 1).

### Visits to an alternative medical practitioner

We did not find significant associations for men between visits to alternative medical practitioners and any of the three included alcohol consumption variables (Table 2). For women, we found that individuals who had experienced injuries because of their drinking had 1.69 times higher odds (95% CI 1.16–2.47) to have applied an alternative medical practitioner compared to those who never had experienced injuries because of drinking (Table 3).

**Table 2:** Association between alcohol and CAM for male participants

	Alternative practitioner^a^				Herbal medicine^b^				Self-treatment^c^			
	Unadjusted		Adjusted		Unadjusted		Adjusted		Unadjusted		Adjusted	
	OR (95% CI)	*P*-value	OR (95% CI)	*P*-value	OR (95% CI)	*P*-vaule	OR (95% CI)	*P*-value	OR (95% CI)	*P*-value	OR (95% CI)	*P*-value
Alcohol Frequency of use												
Never	1.00		1.00		1.00		1.00		1.00		1.00	
Monthly or more infrequently	1.10 (0.79–1.66)	0.648	0.98 (0.63–1.50)	0.911	0.80 (0.60–1.06)	0.117	0.84 (0.62–1.14)	0.272	0.91 (0.41–2.02)	0.818	0.95 (0.40–2.23)	0.900
2–4 times a month	1.05 (0.70–1.56)	0.807	0.96 (0.63–1.45)	0.834	0.81 (0.62–1.06)	0.122	0.94 (0.70–1.26)	0.686	0.81 (0.38–1.75)	0.590	0.73 (0.31–1.68)	0.454
2–3 times a week	0.91 (0.59–1.40)	0.671	0.86 (0.54–1.40)	0.523	0.84 (0.63–1.13)	0.260	1.01 (0.73–1.38)	0.970	1.33 (0.60–2.95)	0.481	1.21 (0.51–2.87)	0.670
More that 3 times a week	0.82 (0.46–1.46)	0.495	0.86 (0.47–1.57)	0.627	1.10 (0.76–1.58)	0.620	1.19 (0.81–1.77)	0.371	1.09 (0.39–3.05)	0.865	1.1 (0.36–3.17)	0.903
Units of alcohol consumed when drinking												
1–2 units	1.00		1.00		1.00		1.00		1.00		1.00	
3–4 units	1.07 (0.85–1.35)	0.560	1.11 (0.87–1.41)	0.404	0.88 (0.74–1.04)	0.127	0.99 (0.83–1.18)	0.880	1.18 (0.76–1.83)	0.462	1.12 (0.72–1.76)	0.615
5 or more units	1.05 (0.78–1.41)	0.732	0.93 (0.67–1.28)	0.662	0.86 (0.69–1.06)	0.162	1.07 (0.84–1.35)	0.590	1.13 (0.64–1.99)	0.680	0.85 (0.46–1.56)	0.602
Injuries because of drinking												
Never	1.00		1.00		1.00		1.00		1.00		1.00	
Yes	1.08 (0.78–1.50)	0.626	0.98 (0.69–1.37)	0.890	1.12 (0.89–1.40)	0.333	1.31 (1.03–1.66)	0.027	1.65 (0.97–2.79)	0.064	1.23 (0.72–2.12)	0.449

^a^ Visited an alternative medical practitioner within the previous year. ^b^ Used herbal or “natural” medicine within the previous year. ^c^ Used meditation, yoga, qi gong or tai-chi as self-treatment within the previous year. Adjusted *p*-value, OR and CI were adjusted for health status (cat.), household income (cat.), age (count) and level of education (cat). Cat.: categorical; Cont.: continuous

**Table 3:** Association between alcohol and CAM for female participants

	Alternative practitioner^a^				Herbal meedicine^b^				Self-treatment^c^			
	Unadjusted		Adjusted		Unadjusted		Adjusted		Unadjusted		Adjusted	
	OR (95% CI)	*P*-value	OR (95% CI)	*P*-value	OR (95% CI)	*P*-value	OR (95% CI)	*P*-value	OR (95% CI)	*P*-value	OR (95% CI)	*P*-value
Alcohol Frequency of use												
Never	1.00		1.00		1.00		1.00		1.00		1.00	
Monthly or more infrequently	0.94 (0.75–1.17)	0.570	0.88 (0.68–1.14)	0.336	1.13 (0.94–1.37)	0.198	1.20 (0.96–1.48)	0.105	2.02 (1.32–3.09)	0.001	1.46 (0.92–2.30)	0.104
2–4 times a month	1.17 (0.94–1.45)	0.161	1.08 (0.84–1.39)	0.555	1.36 (1.14–1.64)	0.001	1.43 (1.15–1.78)	0.001	2.86 (1.89–4.31)	0.000	1.71 (1.09–2.66)	0.019
2–3 times a week	1.04 (0.80–1.34)	0.779	1.02 (0.76–1.37)	0.885	1.30 (1.05–1.61)	0.015	1.37 (1.08–1.75)	0.010	3.69 (2.39–5.69)	0.000	2.07 (1.29–3.31)	0.002
More that 3 times a week	1.09 (0.75–1.58)	0.649	1.13 (0.75–1.71)	0.550	1.60 (1.91–2.16)	0.002	1.76 (1.27–2.44)	0.001	4.16 (2.46–7.03)	0.000	2.62 (1.48–4.61)	0.001
Units of alcohol consumed when drinking												
1–2 units	1.00		1.00		1.00		1.00		1.00		1.00	
3–4 units	1.13 (0.95–1.35)	0.153	1.03 (0.85–1.24)	0.763	1.01 (0.87–1.16)	0.918	1.03 (0.89–1.21)	0.668	1.12 (0.89–1.40)	0.335	0.90 (0.71–1.15)	0.395
5 or more units	1.03 (0.72–1.48)	0.862	0.79 (0.54–1.17)	0.247	0.76 (0.55–1.05)	0.092	0.76 (0.54–1.07)	0.114	1.05 (0.65–1.71)	0.837	0.77 (0.46–1.28)	0.316
Injuries because of drinking												
Never	1.00		1.00		1.00		1.00		1.00		1.00	
Yes	1.94 (1.35–2.79)	0.000	1.69 (1.16–2.47)	0.006	1.45 (1.06–2.00)	0.022	1.38 (0.98–1.93)	0.059	2.85 (1.91–4.24)	0.000	1.95 (1.28–2.96)	0.002

^a^ Visited an alternative medical practitioner within the previous year. ^b^ Used herbal or “natural” medicine within the previous year. ^c^ Used meditation, yoga, qi gong or tai-chi as self-treatment within the previous year. Adjusted *p*-value, OR and CI were adjusted for health status (cat.), household income (cat.), age (count) and level of education (cat). Cat.: categorical; Cont.: continuous

### Use of herbal or “natural” medicine

The odds of using herbal or “natural” medicine were 76% higher (95% CI 1.27–2.44) in women who drank alcohol at least 4 times a week compared to alcohol abstainers (Table 3) The odds for women who drank 2–4 times a month and 2–3 times a week were ORs of 1.43 (95% CI 1.15–1.78) and 1.37 (95% CI 1.08–1.75) respectively compared to teetotallers.

In men, an association was found between the use of herbal or “natural” medicine and injuries caused by drinking. Men who had experienced injuries as a result of their drinking, had a 31% (95% CI 1.03–1.66) higher odds of having applied herbal or “natural” medicine in the previous 12 months compared to those who had not experienced injuries (Table 2). No association was found between the use of herbal or “natural” medicine and other alcohol consumption patterns.

### Used self-treatment techniques

An association was found between use of self-treatment (meditation, yoga, qi gong or tai-chi) and frequency of alcohol consumption for women. The odds of having used such self-treatment techniques were highest among those who drank more than 3 times a week, with an odds ratio of 2.62 (95% CI 1.48–4.61) compared to alcohol abstainers (Table 3). We also found a strong positive association for having used self-treatment techniques for those who reported drinking 2–4 times a month (OR 1.71, 95% CI 1.09–2.66) and 2–3 times a week (OR 2.07, 95% CI 1.29–3.31) compared to teetotallers. The odds of using self-treatment techniques were 96% higher (95% CI 1.28–2.96) in women who reported injuries caused by their drinking compared to those with no such experience. No significant relationship was found between the use of self-treatment techniques and alcohol consumption patterns in men (Table 2).

## Discussion

We found a positive relationship between more frequent alcohol consumption and use of herbal or “natural” medicine and self-treatment techniques in women, but not in men. We also found a positive association between having experienced injuries to themselves or others because of their drinking and CAM use in general among women. For men this association was found only for herbal or “natural” medicine.

### CAM use and alcohol consumption

Studies on CAM use and alcohol consumption are limited and are conducted in few countries. The findings on whether and to what extent alcohol consumption is associated with use of CAM is not consistent [1, 9, 12, 23, 28, 43]. Few studies have gender specific analyses despite the fact that both CAM use and alcohol consumption is influenced by gender [1, 10, 22]. In accordance with the men in the present study, several other studies failed to find any significant association between alcohol consumption and CAM use [30–32]. Ever drinkers were, however found to be more likely to have used CAM, compared to lifetime teetotallers in the US [27]. This is in accordance with our findings among women where ever drinkers of alcohol were more likely to have used herbal medicine and CAM self-treatment than the teetotallers. They found, however that those who drank infrequently (less than one alcohol unit a week) had the highest use of CAM, while heavy drinkers (15 or more units a week) were least likely to have used CAM. This is in contrast to the men in the present study where no significant differences were found, and to the women who were more likely to have used herbal medicine and self-help techniques the more frequently they drank. Grey et al. found that CAM users reported a lower overall consumption of alcohol than non-users [29]. One of the reasons for the inconsistency in our findings compared to the findings in these two studies from the US might be different CAM use and alcohol consumption patterns in the two countries [12, 23, 43]. One possibility is that Norwegian women drink more frequently, but when doing so they drink small amounts. This is suspected as 84% of the women who were drinking alcohol more than 3 times a week reported to only drink 1–2 units of alcohol when drinking.

It might also be that the participants used both CAM and alcohol to cope with the same condition. Partner strain, for instance, have been associated with both increased use of CAM [44] and alcohol [42]. Also pain and psychiatric problems [45–49] could contribute to explain the association between CAM use and alcohol consumption in women. CAM users have shown to be more likely to report mental disorders such as major depression and panic disorders compared to non-users [44] and might also drink alcohol to cope with the same issues. Some CAM therapies have also been used as strategies to cope with alcohol craving and dependencies [45–48], which also could explain the association found in this study. The relationship is complex, as many factors in life could influence both on use of CAM and alcohol consumption.

### CAM use and injuries caused by drinking

This study revealed an association between having experiences injuries caused by own drinking and use of CAM for both men and women. One possible explanation for the association, could be the Norwegian drinking culture that is characterized by heavy episodic drinking during the weekends [4], causing people without drinking problems to injury themselves or others. Injuries caused by drinking and other discomfort caused by heavy drinking could also increase the need for medical treatment and pain relief, thus increase the use of CAM modalities.

### Gender differences

Most of the associations found between CAM modalities and alcohol consumption, was found among the female participants. The only significant association found for men was between use of herbal or “natural” medicine and injuries cause by own drinking. This relationship was, however, not significant for women. The gender differences found are likely due to different associations for use of CAM and different patterns of alcohol consumption for men and women [4, 17, 42, 49]. Men often frame their use of CAM in terms of rationality and have reported treatment of health related issues as their main motivation for CAM use. Women, on the other hand, use in addition CAM to deal with low self-esteem, eating disorders and body image concerns [49]. The association between CAM use and injuries caused by drinking was found only for men. The reason for this might be that men experience such injuries more frequently than women [50]. It is also possible that such drinking behaviour is more accepted among men, leading women to underreport such behaviour.

### Strength and limitations

The main strength of this study is the large number of participants (*n* = 12,981) representing 20% of the total population of Tromsø [51], and the rather high response rate of 65%.

Populations based studies are considered to be an excellent source of data in research [36], nevertheless, the results should be interpreted in light of some limitations. These data reflects a cross-sectional set of associations with no information on possibly causal events [52]. The experience of injuries caused by own drinking was recoded into ever having had such an experience while the question of CAM use was restricted to use within the last 12 months. Injuries caused by drinking might have happened only once and/or a long time ago and might not be representative for that person’s current or general alcohol consumption. Another limitation is that the findings are based on self-reported data that might be influences by the participants’ perceptions of right and wrong and misinterpretations of the questions [52, 53]. Both intentionally and unintentionally, people tend to overestimate their healthy lifestyle choices and underestimate unhealthy habits [52]. Hence, questions regarding alcohol consumption and injuries caused by drinking could be especially prone to report bias [53, 54].

The ability to answer accurately and completely could be difficult when describing drinking behaviour in distant past [53]. Injuries might also occur under severe intoxication, when blackouts are not uncommon [55] and it is therefore likely to be under-reported.

Reduced accuracy due to recall bias might also be present for CAM, as participants were asked to report use as far back as 12 months. Men might also be more prone to underreport use of CAM compared to women as CAM use is often associated with feminine qualities and traditional female gender roles [22]. Women on the other hand, might be less inclined to report heavy episodic drinking and injuries caused by drinking due to the same traditional gender roles.

Due to the fact that CAM users often apply more than one CAM approach, the different CAM variables were not mutually exclusive in the analyses. The non-users of one approach might still have applied other CAM modalities. The analyses does therefor not compare CAM users to non-users of CAM in general. This is in line with the aim of the study which was to compare users of the different CAM approaches to non-users of these. Finally, even though we have adjusted for the most important factors, a residual confounding cannot be excluded.

### Implication of the findings

The main aim of this study was to address the almost total lack of studies investigating the associations between alcohol consumption and use of different CAM approaches. Knowledge of this association could be important for health care personnel when discussing the patient’s health problems and how the patient deal with these problems themselves, and further how approaches like CAM use and alcohol consumption can interact with conventional care. CAM providers can use the findings of this study to discuss their client’s use of alcohol and risks of excessive drinking and further, to suggest other, healthier ways to cope with the cause of their drinking.

### Future research

The findings from this study cannot fully explain the relationship between alcohol consumption and CAM approaches, and inconsistency in international findings indicate that both CAM and alcohol use vary across cultures and over time. The relationship is likely to be complex, as many factors in life could influence both use of CAM and alcohol consumption. In order to get a clearer picture of the associations between CAM use and alcohol consumption, further research is needed focusing on the underlying causes of use of different CAM modalities and alcohol consumption patterns. There is also a need for research with longitudinal design to explore the causation of the relationship.

## Conclusion

In this study we found different associations between CAM use and alcohol consumption than what is found earlier in studies conducted in other countries. This underline the need for local studies since both patterns of CAM use and alcohol consumption varies widely across cultures and regions. The associations between frequent alcohol consumption and injuries caused by drinking and use of CAM in Norway can be useful for both conventional and unconventional health care personnel in meeting with their patients.

## Abbreviations

€: Euro
CAM: Complementary and alternative medicine
CI: Confidence intervals
NOK: Norwegian kroner
OR: Odds ratio
REK: Regional Committee of Medical and Health Research Ethics
UiT: University of Tromsø
